# Prevalence of MTHFR C677T polymorphism and its association with serum homocysteine and blood pressure among different ethnic groups: insights from a cohort study of Nepal

**DOI:** 10.1186/s12872-025-04690-z

**Published:** 2025-03-29

**Authors:** Rajendra Dev Bhatt, Biraj Man Karmacharya, Archana Shrestha, Dinesh Timalsena, Surendra Madhup, Rajesh Shahi, Nishan Katuwal, Rajeev Shrestha, Annette L. Fitzpatrick, Prabodh Risal

**Affiliations:** 1 Department of Clinical Biochemistry, Dhulikhel Hospital-Kathmandu University Hospital, Dhulikhel, Nepal; 2 Department of Public Health, Dhulikhel Hospital, Kathmandu University Hospital, Dhulikhel, Nepal; 3 Department of Microbiology, Dhulikhel Hospital, Kathmandu University Hospital, Dhulikhel, Nepal; 4 Department of Molecular Biology, Dhulikhel Hospital, Kathmandu University Hospital, Dhulikhel, Nepal; 5 Department of Pharmacology, Dhulikhel Hospital, Kathmandu University Hospital, Dhulikhel, Nepal; 6 Departments of Family Medicine and Epidemiology, Schools of Medicine and Public Health, University of Washington, Seattle, USA

**Keywords:** Hyperhomocysteinemia, Hypertension, MTHFR C677T, Nepal, Polymorphism

## Abstract

**Background:**

The risk of hypertension varies based on ethnicity, environmental factors, and genetic predispositions. Studies have reported a higher risk of cardiovascular diseases (CVD) and hypertension among the *Newar* ethnic groups in Nepal. However, the genetic analysis for Methylenetetrahydrofolate reductase (MTHFR C677T) gene mutations, serum homocysteine, and high-sensitivity C-reactive protein (hs-CRP) levels across different ethnicities remains unexplored.

**Methods:**

Sociodemographic information and baseline data of 489 participants were obtained from the first phase of the Dhulikhel Heart Study. Preserved blood samples were analyzed for MTHFR C677T polymorphism using real-time polymerase chain reaction (TaqMan assay), and serum homocysteine was measured through immunoassay techniques. Descriptive analysis, the Hardy-Weinberg equilibrium test, and multinomial regression were performed.

**Results:**

The prevalence of homozygous mutation (TT) was 19.8% in the *Newar* group and 12.5% in the *Brahmin/Chhetri* ethnicity. The highest mean value of homocysteine (19.4 µmol/L) was observed in homozygous participants, followed by the heterozygous mutant group (17.4 µmol/L). A statistically significant association (*P* = < 0.001) was found between homocysteine levels and blood pressure.

**Conclusions:**

The Dhulikhel Heart Study reveals a significant prevalence of the MTHFR C677T gene mutation among the *Newar* ethnicity compared to other groups. Elevated levels of homocysteine and high-sensitivity C-reactive protein (hs-CRP) were associated with increased blood pressure.

**Clinical trial number:**

Not applicable

## Introduction

Essential hypertension (EH) is a complicated condition that most likely arises from the interplay of several gene-environmental interaction and their identification is essential for the prevention and management of cardiovascular diseases (CVD) [1, 2]. Various environmental and clinical risk factors are linked to essential hypertension, such as high sodium intake, alcohol consumption, lack of physical activity, poor diet, obesity, insulin-resistant diabetes, and hyperlipidemia. While these factors significantly contribute to hypertension susceptibility, it is estimated that genetic factors account for up to 60% of the variation in hypertension risk [3–5].

Genome-wide association studies (GWASs) have identified common genetic variations linked to both normal and pathological traits, indicating that single nucleotide polymorphisms (SNPs) can serve as effective biomarkers for screening at-risk populations and facilitating genetic diagnosis and gene therapy for hypertension [6].

Methylenetetrahydrofolate reductase (MTHFR) is an enzyme encoded by the MTHFR gene and is responsible for homocysteine (Hcy) and folate metabolism [7]. According to a metanalysis of case-control studies, the C677T polymorphism in MTHFR is linked to 36–87% higher incidence of hypertension due to reducing catalytic activity by MTHFR enzyme resulting in hyperhomocysteinemia (HHcy) [8]. This C677T polymorphism occurs in exon 4, where cytosine is replaced by thymine at the 677th position, leads to the replacement of Alanine with Valine at position 222 in the protein (A222V) [9]. Hyperhomocysteinemia, induced by this genetic variation, is considered an independent risk factor for cardiovascular diseases (CVD), stroke, and venous thrombosis, which triggers the proliferation of vascular smooth muscle and endothelial injury and contributes to hypertension [10].

Currently, an estimated 1.28 billion people suffer from hypertension, and this number is projected to escalate to 1.56 billion by 2025 [11]. In the context of Nepal, hypertension affects 27.3% of the general population, with a notable increase to 31.3% in the age group of ≥ 35 years [12]. The prevalence of hypertension in the Nepalese population varies based on ethnicity and geography, with the lowest rates observed in mountainous regions and the highest in hilly regions. Gandaki Province ranks on the top with a prevalence of 27.5%, followed by Bagmati Province 24.1% and lowest (13.9%) in Far Western Province [13].

Nepal is currently undergoing rapid transformations in diet, demography, and epidemiology, potentially exerting a substantial impact on the distribution and risk of non-communicable diseases [14, 15]. In Dhulikhel, Nepal, a community-based longitudinal cohort study revealed that individuals belonging to the *Newar* ethnic group faced a 5.6 times higher risk of hypertension attributable to disparities in dietary practices and other environmental factors. Numerous studies from Nepal indicated that 51-64.5% of patients with a history of hypertension or under medication struggle with inadequate blood pressure control [16, 17]. However, the genetic association behind the higher risk of hypertension and poor blood control among under medication patients remains elusive. Earlier meta-analyses have suggested a link between the MTHFR C677T polymorphism and a heightened risk of coronary artery disease [18, 19] and high levels of plasma homocysteine have been observed in hypertensive patients and are positively correlated with blood pressure and ischemic stroke [20]. A two-year, randomized, placebo-controlled trial demonstrated that participants assigned to homocysteine-lowering treatment experienced a reduction in blood pressure [21].

All these findings originated from developed countries or urban areas, but there was a lack of similar evidence among the Nepalese ethnic groups. This research could aid in identifying target populations of CVD and hypertension and formulating affordable treatment plans tailored to the diverse needs of ethnic groups with different genetic mutations on MTHFR C677T and levels of serum homocysteine for the prevention and management of hypertension.

Hence, the primary objective of this study was to investigate the prevalence of polymorphisms in the MTHFR C677T gene, assess plasma homocysteine levels and high-sensitive C-reactive protein (hs-CRP) in well-preserved blood specimens of Dhulikhel Heart Study participants, and examine their association with blood pressure.

## Materials and methods

The Dhulikhel Heart Study (DHS) was conducted in 2013–2015 as cohort study in Dhulikhel municipality, Nepal aimed to investigate cardiovascular risk factors within a representative population. In the first phase, one third households of municipality were randomly selected and comprehensive baseline data were collected. Every household was invited to participate in the survey, including all eligible adults aged 18 or older who had resided there for at least six months. The study excluded pregnant women, individuals who declined to provide consent, those residing in institutionalized settings (such as hostels and hotels), and those facing communication difficulties due to medical or mental conditions [16, 17].

Building on this foundation, the second phase of the study adopted a cross-sectional design utilizing preserved blood samples from the initial cohort in 2023.The baseline data for this study were derived from the initial phase of the Dhulikhel Heart Study (DHS). A well-preserved whole blood and serum sample (stored at -80^o^C) from 535 DHS participants were used for the analysis of the MTHFR C677T gene and homocysteine. However, only the results from 489 samples were deemed suitable for inclusion in this study because 27 blood sample had poor labelling, 19 were insufficient for analysis. Ethical approval for the study was granted by the Institutional Review Committee of Kathmandu University School of Medical Science (IRC-KUSMS), with approval number 35/23.

### Genotyping and DNA extraction

DNA extraction from whole blood was carried out using a DNA extraction kit (Zymo Research Inc., Catalogue Number: D4068) [22]. Subsequently, qPCR was performed using the molecular genetics MTHFR (C677T) kit (Sacace Biotechnologies, Catalogue Number: T01103-5-T) [23] to identify mutations in the MTHFR C677T gene, utilizing the CFX96 Bio-Rad system (BioRad Laboratories). The entire process of extraction, elution, and analysis adhered to the manufacturer’s instructions. For the quantitative determination of homocysteine, a chemiluminescent microparticle immunoassay (CMIA) [24] was employed, while hs-CRP was quantified using a fully automated biochemistry analyzer (BA 400, Biosystems, Spain). Both analyses were conducted according to the manufacturer’s guidelines [25].

### Interpretation of results

Following the completion of qPCR, (TaqMan assay) the baseline was established within cycles 3–15, and the threshold was set at 100. Regarding the results, if the sample exhibited signals for HEX, FAM, HEX, and FAM, both polymorphisms were interpreted as homozygous wild type, homozygous mutated, and heterozygous mutated, respectively. Samples with a Ct value > 32 was deemed invalid and subjected to qPCR repetition. If the results remained invalid in the second run, they were subsequently excluded [23]. Genotyping quality was assured by re-genotyping a randomly selected subset of 10% of the total samples. Additionally, standard quality control including (positive and negative controls) was run in each PCR run to minimize technical errors. The distribution of hs-CRP serum concentrations was classified into three groups. Cardiac risk was deemed low, average, and high when the hs-CRP value was < 1.0 mg/L, 1.0–3.0 mg/L, and > 3.0 mg/L, respectively [26–29]. Serum homocysteine level < 15.0 µmol/L considered normal while 15–30 µmol/L, 30–100 µmol/L and > 100.0 µmol/L is moderate, intermediate and severe high respectively [30].

### Statistical analysis

Continuous variables were presented as mean and standard deviation, while categorical variables were expressed as frequency and proportions using SPSS version 23.0. The Hardy-Weinberg equilibrium test was employed to calculate the Chi-square (χ²) value, assessing the concordance between the observed and expected frequencies of different alleles. Descriptive analysis, one-way analysis of variance (ANOVA), and bivariate correlation were utilized to examine mean differences for continuous variables across categorical variables. Statistical significance was established when the *P*-value was < 0.05.

## Results

Among all the participants, 60.5% were female, with the highest representation from the *Newar* ethnic group (39.1%), followed by *Brahmin/Chhetri* (35.8%) and *Tamang* (18.4%). The average age was 41.1 (± 16.2) years. Over one-third (37.2%) of the participants were overweight, while 8.4% were obese. Additionally, 29.7% had hypertension, and 33.0% were pre-hypertensive (Table 1).

**Table 1:** Baseline characteristics of the study population (*n* = 489)

	Minimum	Maximum	Mean	Std. Deviation
Age (Years)	18.0	85.0	41.1	16.2
FBG (mg/dL)	38.0	324.0	79.2	19.1
Total Cholesterol (mg/dL)	89.0	352.0	166.7	41.0
HDL Cholesterol (mg/dL)	21.0	91.0	42.3	11.4
LDL Cholesterol (mg/dL)	45.0	231.0	108.3	29.3
Triacylglycerol (mg/dL)	42.0	674.0	125.0	73.8
HbA1C (%)	3.9	12.0	6.1	0.9
Homocysteine (µmol/L)	1.4	50.0	16.1	10.7
hs-CRP (mg/L)	0.10	13.5	1.8	1.3
Systolic BP (mmHg)	87.0	197.0	128.2	18.3
Diastolic BP (mmHg)	55.0	125.0	82.0	10.5
Pulse	53.0	121.0	78.4	10.6
Height (cm)	130.0	186.0	156.0	8.9
Weight (kg)	39.0	95.0	59.9	10.8
Waist (cm)	56.0	121.0	82.2	11.0
Hip (cm) BMI	60.0 15.6	129.0 39.0	91.4 24.5	8.9 3.9

^*^FBG = Fasting Blood Glucose, HDL = High Density Lipoprotein, LDL = Low Density Lipoprotein

HbA1c = Glycated hemoglobin, hs-CRP = high sensitive C reactive protein, BMI = body mass index

The Hardy-Weinberg equilibrium test identified 227 CC genotypes, 187 CT heterozygotes, and 75 TT homozygous mutants (Table 2). Applying Hardy-Weinberg equilibrium based on the expected values resulted in a statistically significant Chi-square value of 11.59 (*P* = 0.003). The overall prevalence of homozygous (TT) mutations was 15.3%, while that of heterozygous (CT) polymorphisms was 38.2%.

**Table 2:** Hardy–Weinberg equilibrium test for the MTHFR polymorphisms

MTHFR C677T	Observed Number	Expected Number	Chi-Square (X^2^)	*P*-Value
CC	227	210	11.59	0.003
CT	187	221		
TT	75	58		
Total	489	489		

^*^CC = normal genotype, CT = heterozygous mutant and TT = homozygous mutant

Table 3 displays the descriptive analysis assessing the prevalence of different genotypes across various ethnic groups and genders. The incidence of homozygous mutations (TT) in the *Newar* ethnic group was 7.3% higher than in the *Brahmin and Chhetri* ethnic groups.

**Table 3:** Genotype and allele frequency of different ethnic groups and sex (*n* = 489)

	Genotype CC (*n* = 227) 46.4%	Genotype CT (*n* = 187) 38.2%	Genotype TT (*n* = 75) 15.3%
**Ethnic group**			
*Brahmin and Chhetri* (175)	95 (54.2%)	58 (33.1%)	22 (12.5%)
*Newar* (191)	73 (38.2%)	80 (41.8%)	38 (19.8%)
*Tamang* (90)	47 (52.2%)	35 (38.8%)	8 (8.8.6%)
Others (33)	12 (36.3%)	14 (42.4%)	7 (21.2%)
**Sex**			
Female (296)	145 (48.9%)	106 (35.8%)	45 (15.2%)
Male (193)	82 (42.4%)	81 (41.9%)	30 (15.5%)

^*^CC = normal genotype, CT = heterozygous mutant, and TT = homozygous mutant

Although a notable difference was noted in low-density lipoprotein levels and diastolic blood pressure, this difference was not statistically significant (Table 4). However, the serum homocysteine level exhibited statistically significant (*P* = 0.01).

**Table 4:** Characteristics of the biochemical parameters with respective genotypes (ANOVA)

Biochemical Parameters	Genotype	Mean	Std. Deviation	Significance (*P* = < 0.05)
FBG (mg/dL)	CC Genotype (Normal) CT Genotype (Heterozygous mutant) TT Genotype (Homozygous mutant)	78.6 78.7 82.0	21.3 14.9 20.9	0.38
Total Cholesterol (mg/dL)	CC Genotype (Normal) CT Genotype (Heterozygous mutant) TT Genotype (Homozygous mutant)	163.9 166.5 175.8	39.9 38.5 49.1	0.93
HDL Cholesterol (mg/dL)	CC Genotype (Normal) CT Genotype (Heterozygous mutant) TT Genotype (Homozygous mutant)	42.7 42.4 41.0	11.6 11.1 11.9	0.54
LDL Cholesterol (mg/dL)	CC Genotype (Normal) CT Genotype (Heterozygous mutant) TT Genotype (Homozygous mutant)	106.2 107.0 118.2	27.6 27.3 36.7	0.06
Triacylglycerol (mg/dL)	CC Genotype (Normal) CT Genotype (Heterozygous mutant) TT Genotype (Homozygous mutant)	122.7 124.8 132.7	74.3 68.7 84.2	0.59
HbA1C (%)	CC Genotype (Normal) CT Genotype (Heterozygous mutant) TT Genotype (Homozygous mutant)	6.1 6.0 6.3	0.9 0.8 1.1	0.05
Homocysteine (µmol/L)	CC Genotype (Normal) CT Genotype (Heterozygous mutant) TT Genotype (Homozygous mutant)	13.9 17.4 19.4	8.8 11.9 11.4	0.01
hs-CRP (mg/L)	CC Genotype (Normal) CT Genotype (Heterozygous mutant) TT Genotype (Homozygous mutant)	1.7 1.9 2.0	1.2 1.3 1.4	0.16
Systolic BP (mmHg)	CC Genotype (Normal) CT Genotype (Heterozygous mutant) TT Genotype (Homozygous mutant)	126.1 129.1 132.3	18.5 17.8 18.2	0.27
Diastolic BP (mmHg)	CC Genotype (Normal) CT Genotype (Heterozygous mutant) TT Genotype (Homozygous mutant)	81.0 82.5 83.8	10.2 11.2 9.6	0.09

^*^FBG = Fasting blood glucose, HDL = High Density Lipoprotein, LDL = Low Density Lipoprotein

HbA1c = Glycated hemoglobin, hs-CRP = high sensitive C reactive protein, BP = blood pressure

In the normal group (no mutation on MTHFR C677T), the mean serum homocysteine level was 13.9 ± 8.8 (µmol/L), while the heterozygous and homozygous mutant groups exhibited levels of 17.4 ± 11.9 and 19.4 ± 11.4 (µmol/L), respectively. A positive Pearson correlation was observed between serum homocysteine levels and systolic blood pressure (*r* = 0.18) as well as diastolic blood pressure (*r* = 0.19). Furthermore, there was a positive correlation between homocysteine and hs-CRP (*r* = 0.47, *P* = < 0.001), Total cholesterol (*r* = 0.23, *P* = < 0.001), and LDL cholesterol (*r* = 0.20, *P* = < 0.001).

The outcomes of a multivariate multinomial logistic regression analysis (Table 5) revealed significant associations between various factors and distinct genotype categories, even after adjusting for age, sex, ethnicity, hypertension, homocysteine levels, and lipid profiles. In comparison to *Brahmin/Chhetri*, individuals of *Newar* ethnicity exhibited a 75% increase in the relative risk for Alleles 1 and 2 and heterozygous mutant compared to Allele 1 and homozygous wild type (RR = 1.75, 95% CI: 1.09–2.81, *P* = 0.02). Furthermore, participants with hypertension were more likely to fall into the Allele 2 homozygous mutant category (RR = 2.28, 95% CI: 1.15–4.48, *P* = 0.01), while keeping other variables in the model constant.

**Table 5:** Multinomial logistic regression analysis to calculate factors associated with the genotype (*n* = 489)

	Allele 1 (CC) (Reference)	Allele 2 (TT) homozygous mutants			Allele 1 and 2 (CT) heterozygous mutants		
	RR*	RR	95% CI	*P*-value	RR	95% CI	*P*-value
Age in years	1	1.00	0.97–1.01	0.72	1.00	0.98–1.01	0.61
Sex Male	1	0.86	0.47–1.54	0.60	1.28	0.84–1.94	0.25
**Ethnicity**							
*Tamang*	1	0.81	0.32–1.20	0.64	1.21	0.68–2.11	0.51
*Newar*	1	1.87	0.98–3.56	0.06	1.75	1.09–2.81	0.02
Others	1	2.55	0.86–7.48	0.08	1.77	0.75–4.15	0.19
Hypertension	1	2.28	1.15–4.48	0.01	1.45	0.84–2.48	0.18
Homocysteine	1	1.04	1.01–1.06	< 0.05	1.03	1.00-1.05	0.01
HDL	1	0.97	0.94–0.99	0.03	1.00	0.97–1.01	0.63
LDL	1	1.01	1.00-1.02	0.03	1.00	0.99-1.00	0.61

*RR stands for relative risk ratio. Brahmin/Chhetri ethnicity was used as the reference group for the ethnicity variable. Bold *p*-values indicate significance at the 5% level

## Discussion

This study pioneers the demonstration of polymorphisms in the MTHFR C677T gene across various ethnic groups and their association with homocysteine and blood pressure in the participants of this cohort of Dhulikhel municipality and holds significance on two fronts. Firstly, it affirms that the extent of polymorphism in the MTHFR C677T gene varies among representative population of a suburban community of Nepal. Secondly, it establishes an association between the mutation of the MTHFR gene and hyperhomocysteinemia, as well as high blood pressure, particularly in the *Newar* ethnic groups in a sub-urban community in central Nepal. The genetic mutation causes decreased activity of MTHFR activity, increased plasma homocysteine and MTHFR activity in carriers of CT and TT genotype is 35% and 70% lower than in normal subjects respectively [31]. Although elevated homocysteine is a risk of cardiovascular illness, its connection to hypertension is still up for debate but studies has clearly established causal relationship with blood pressure and HHcy [32].

Our analysis revealed a notable prevalence of the homozygous TT genotype among the Newar group (19.8%), significantly higher than in the *Brahmin/Chhetri* group (12.5%). This suggests a genetic predisposition that might contribute to the higher incidence of hypertension observed in the *Newar* community. Analysis of the Hardy–Weinberg equilibrium indicated a notable deviation in the genotype frequencies for the MTHFR C677T polymorphism (χ² = 11.59, *P* = 0.003). The observed genotype frequencies for CC, CT, and TT (227, 187, and 75, respectively) were different from the expected frequencies (210, 221, and 58, respectively). The mutations in the TT allele in our study closely resemble those observed in Europe, North America, and some Asian countries. In Europe and North America, the prevalence of the TT homozygous genotype ranges from 8 to 18%, though it is notably lower in individuals with dark skin (1.45%) [33]. The frequency of the TT genotype is 15% among the Japanese, 14% among Koreans, 8-32.8% among Chinese, and 0-3.7% among Indian populations [34]. The results indicate a possible genetic predisposition or evolutionary impact on the distribution of the MTHFR C677T polymorphism within this population.

The average homocysteine level in our study was 16.1 µmol/L, and the frequency of polymorphisms, particularly in the homozygous state (TT), was found to be comparatively higher than in other similar studies [35, 36]. This clearly indicates there is variation in polymorphisms in the MTHFR C677T gene and fluctuations in homocysteine levels among diverse ethnicities. And Homocysteine (Hcy), a demethylated derivative of methionine, could be the possible metabolic cause, recognized as a thrombogenic non-proteinogenic amino acid and is produced within the “1-carbon metabolism (methionine-Hcy-folate)” cycle [36].

The synthesis and removal of Hcy typically maintain a delicate balance, but in pathological states, such as hyperhomocysteinemia (HHcy), overall plasma Hcy levels tend to rise due to impaired Hcy metabolism. The primary causes of HHcy in individuals include: (1) regular consumption of an excessive methionine-rich protein diet; (2) insufficiency of vitamin B12 and/or folate; (3) the presence of heterozygous or homozygous cystathionine β-synthase (CBS) gene; and (4) insufficient clearance of Hcy from the kidney [37].

In our investigation, we identified a higher level of homocysteine (Hcy) in the *Newar* ethnic groups compared to other groups and a distinct dietary pattern, incorporating refined grains, meat, and alcohol as major components in their cuisine [17] could be the possible link. The dietary intake of methionine influences the rate of S-adenosylmethionine (SAM) production, which, in turn, could impact the homocysteine pathway [38].

These findings suggested that there was high prevalence of MTHFR C677T gene polymorphism and high level of homocysteine associated with high blood pressure in participated population and varies with ethnic groups. In spite of these genetic and biochemical association with blood pressure, finding cannot be generalized for entire Nepalese population. So bigger sample size and multicenter study are recommended to establish more clear findings to address genetic and biochemical risk factors which could enhance the effectiveness of hypertension prevention and treatment programs for different ethnic groups in Nepal.

## Conclusions

This cohort study highlights high prevalence of the MTHFR C677T gene mutation among selected ethnic groups. The *Newar* ethnic group exhibited the highest mutation frequency and TT homozygous genotype showed the strongest association with elevated serum homocysteine and increased blood pressure.

## Data Availability

The data supporting this study’s findings are available upon reasonable request from Dhulikhel Hospital-Kathmandu University Hospital or corresponding author. However, due to ethical guidelines and confidentiality agreements, raw participant data cannot be shared. Aggregated and anonymized data may be provided after obtaining approval from the relevant institutional and ethical review board.

## Abbreviations

ANOVA: Analysis of variance
BMI: Body mass index
BP: Blood pressure
CMIA: Chemiluminescent microparticle immunoassay
CVDs: Cardiovascular disease (s)
DHS: Dhulikhel heart study
FBG: Fasting blood glucose
Hcy: Homocysteine
HDL: High density lipoprotein
HHcy: Hyperhomocysteinemia
hs-CRP: High sensitive C reactive protein
IRC-KUSMS: Intuitional review committee-Kathmandu university school of medical sciences
LDL: Low density lipoprotein
LMICs: Low- and middle-income country (s)
MTHFR: Methylenetetrahydrofolate reductase
qPCR: Quantitative polymerase chain reaction
SPSS: Statistical package for social sciences
